# 60 Safety of Maintaining Nutrition Through Moderate Sedation for Burn Wound Care

**DOI:** 10.1093/jbcr/irae036.052

**Published:** 2024-04-17

**Authors:** Amber D Kohler, Melanie Donnelly, Blaire Balstad, Scott W Mueller, Lacey Patton, Cameron Gibson, Arek J Wiktor

**Affiliations:** UCHealth Burn & Frostbite Center, Denver, Colorado; Medical College of Wisconsin, Hartland, Wisconsin; University of Colorado Anschtuz, Denver, Colorado; UCHealth and University of Colorado School of Pharmacy, Aurora, CO; University of Colorado Hospital, Littleton, CO; University of Colorado, Aurora, CO; University of Colorado Hospital, Aurora, CO; UCHealth Burn & Frostbite Center, Denver, Colorado; Medical College of Wisconsin, Hartland, Wisconsin; University of Colorado Anschtuz, Denver, Colorado; UCHealth and University of Colorado School of Pharmacy, Aurora, CO; University of Colorado Hospital, Littleton, CO; University of Colorado, Aurora, CO; University of Colorado Hospital, Aurora, CO; UCHealth Burn & Frostbite Center, Denver, Colorado; Medical College of Wisconsin, Hartland, Wisconsin; University of Colorado Anschtuz, Denver, Colorado; UCHealth and University of Colorado School of Pharmacy, Aurora, CO; University of Colorado Hospital, Littleton, CO; University of Colorado, Aurora, CO; University of Colorado Hospital, Aurora, CO; UCHealth Burn & Frostbite Center, Denver, Colorado; Medical College of Wisconsin, Hartland, Wisconsin; University of Colorado Anschtuz, Denver, Colorado; UCHealth and University of Colorado School of Pharmacy, Aurora, CO; University of Colorado Hospital, Littleton, CO; University of Colorado, Aurora, CO; University of Colorado Hospital, Aurora, CO; UCHealth Burn & Frostbite Center, Denver, Colorado; Medical College of Wisconsin, Hartland, Wisconsin; University of Colorado Anschtuz, Denver, Colorado; UCHealth and University of Colorado School of Pharmacy, Aurora, CO; University of Colorado Hospital, Littleton, CO; University of Colorado, Aurora, CO; University of Colorado Hospital, Aurora, CO; UCHealth Burn & Frostbite Center, Denver, Colorado; Medical College of Wisconsin, Hartland, Wisconsin; University of Colorado Anschtuz, Denver, Colorado; UCHealth and University of Colorado School of Pharmacy, Aurora, CO; University of Colorado Hospital, Littleton, CO; University of Colorado, Aurora, CO; University of Colorado Hospital, Aurora, CO; UCHealth Burn & Frostbite Center, Denver, Colorado; Medical College of Wisconsin, Hartland, Wisconsin; University of Colorado Anschtuz, Denver, Colorado; UCHealth and University of Colorado School of Pharmacy, Aurora, CO; University of Colorado Hospital, Littleton, CO; University of Colorado, Aurora, CO; University of Colorado Hospital, Aurora, CO

## Abstract

**Introduction:**

Physiologic changes in burn patients create a hypermetabolic state increasing nutrition requirements. The American Society of Anesthesiology recommends patients be made NPO with enough time to allow for gastric emptying prior to procedural sedation. Daily burn wound care needs requiring sedation make this approach untenable. We aimed to assess the risk of moderate sedation in those without interrupted nutrition.

**Methods:**

A 12-month single centered retrospective analysis at our ABA verified center was performed. Burn patients with natural airways (non-intubated/non-trached) receiving moderate sedation for wound care with a Richmond Agitation-Sedation Score (RASS) goal of -3 were included. Timing of nutritional intake prior to the start of procedural sedation, total amount of drugs administered, lowest RASS score, and all adverse events throughout procedural sedations, including aspiration/pneumonia were recorded. Confidence intervals (CI) were calculated to estimate the risk of adverse events in this cohort.

**Results:**

A total of 97 moderate sedations were completed on 24 patients (66.7% male) with a mean age 43 years (range 18-79), mean total TBSA 13.3% (range 4.5-38.5), and mean ASA score 1.9 (range 1-3). Patients averaged 2.9 oral premedication agents (opiate 100%, lorazepam 92%, ketamine 75%, dexmedetomidine 18%) and 2.75 IV agents administered, with mean doses of fentanyl 148mcg, midazolam 2mg, ketamine 50mg, and maximum dexmedetomidine infusion of 0.85 mcg/kg/hr. Patients achieved a mean RASS of -1.9 (range 0 to -4). On average, nutrition of any kind was consumed 109 minutes prior to sedations, with an average of 171 minutes from consumption of solids. In 24 sedations (24%), 6 patients had tube feeds continued throughout wound care. Location of feeding tubes during sedations: 21 gastric, 3 post-pyloric. One episode of nausea occurred after premedications were given (95% CI 0.03-5.6%). Ondansetron was given to the patient prior to the start of the moderate sedation without further nausea/complications. No complications related to aspiration were noted in any sedation event (97.5% upper CI 3.7%), including no episodes of vomiting, evidence of aspiration, pneumonia, hypoxia, complete airway obstruction, laryngospasm, or death. Hypertension was noted in 17 sedations, treated in 1 event. Hypotension was noted in 6 sedations, each requiring a decrease in the IV dexmedetomidine infusion.

**Conclusions:**

No harmful adverse events were associated with maintained nutrition throughout the peri- and intra-sedative wound care periods, despite ongoing gastric tube feeds in this small sample of burn patients. A large prospective study is required to further validate this practice.

**Applicability of Research to Practice:**

Institutions should consider ongoing nutrition requirements and apply risk-benefit conversations when adopting a burn specific sedation policy.

